# Supplementary material for: The Ninhydrin Reaction Revisited: Optimisation and Application for Quantification of Free Amino Acids
Source: Molecules. 2024 Jul 10;29(14):3262. doi: 10.3390/molecules29143262 (PMC11278723; doi:10.3390/molecules29143262)
Supplement: Supplementary file 1 [file molecules-29-03262-s001.zip › Supplementary Figure S1.pdf]

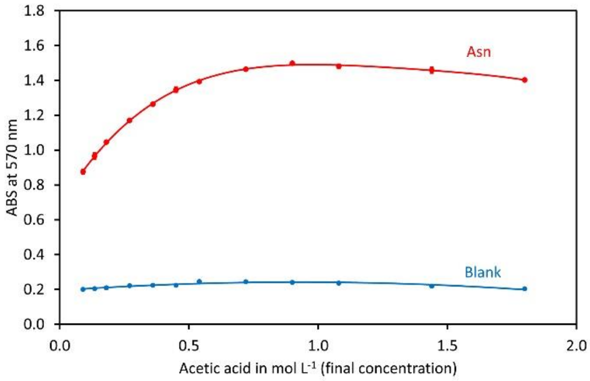

Supplementary Figure S1: Impact of the acetic acid to lithium acetate ratio on the ninhydrin reaction. A concentration of 0.5 mol L^{-1} lithium acetate was used in all reactions while the indicated concentration of acetic acid was set by the addition of glacial acetic acid. All reactions contained 37.5% (v/v) DMSO, 10 g L^{-1} ninhydrin, and 0.375 g L^{-1} hydrindantin. Reactions with asparagine contained 0.5 mmol L^{-1} of the amino acid. All concentrations refer to the final concentrations in the reaction. The reactions were heated to 100°C for 15 min. The data points and error bars represent the averages and standard deviations of four independent reactions, respectively.
