# Supplementary material for: The Ninhydrin Reaction Revisited: Optimisation and Application for Quantification of Free Amino Acids
Source: Molecules. 2024 Jul 10;29(14):3262. doi: 10.3390/molecules29143262 (PMC11278723; doi:10.3390/molecules29143262)
Supplement: Supplementary file 1 [file molecules-29-03262-s001.zip › Supplementary Figure S2.pdf]

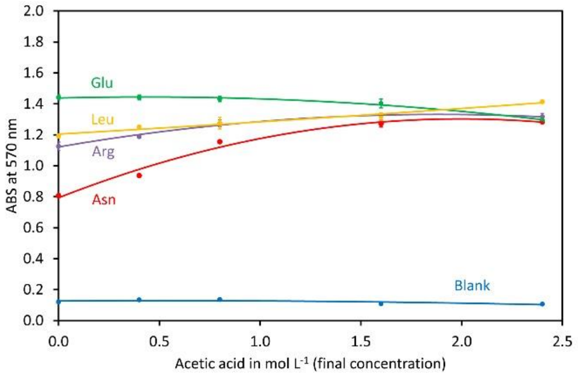

Supplementary Figure S2: Impact of the acetic acid to potassium acetate ratio on the ninhydrin reaction of different amino acids. A final concentration of 0.8 mol L^{-1} potassium acetate was used in all reactions while the indicated concentration of acetic acid was set by the addition of glacial acetic acid to the indicated level. All reactions contained 20 g L^{-1} ninhydrin and 0.4 g L^{-1} hydrindantin dissolved in DMSO/aqueous acetic acid-potassium acetate buffer = 40/60 (v/v). The indicated amino acid was added to a final concentration of 0.4 mmol L^{-1}. The reactions were heated to 90°C for 45 min. The data points and error bars represent the averages and standard deviations of four independent reactions, respectively.
