# Supplementary material for: The Ninhydrin Reaction Revisited: Optimisation and Application for Quantification of Free Amino Acids
Source: Molecules. 2024 Jul 10;29(14):3262. doi: 10.3390/molecules29143262 (PMC11278723; doi:10.3390/molecules29143262)
Supplement: Supplementary file 1 [file molecules-29-03262-s001.zip › Supplementary Figure S3.pdf]

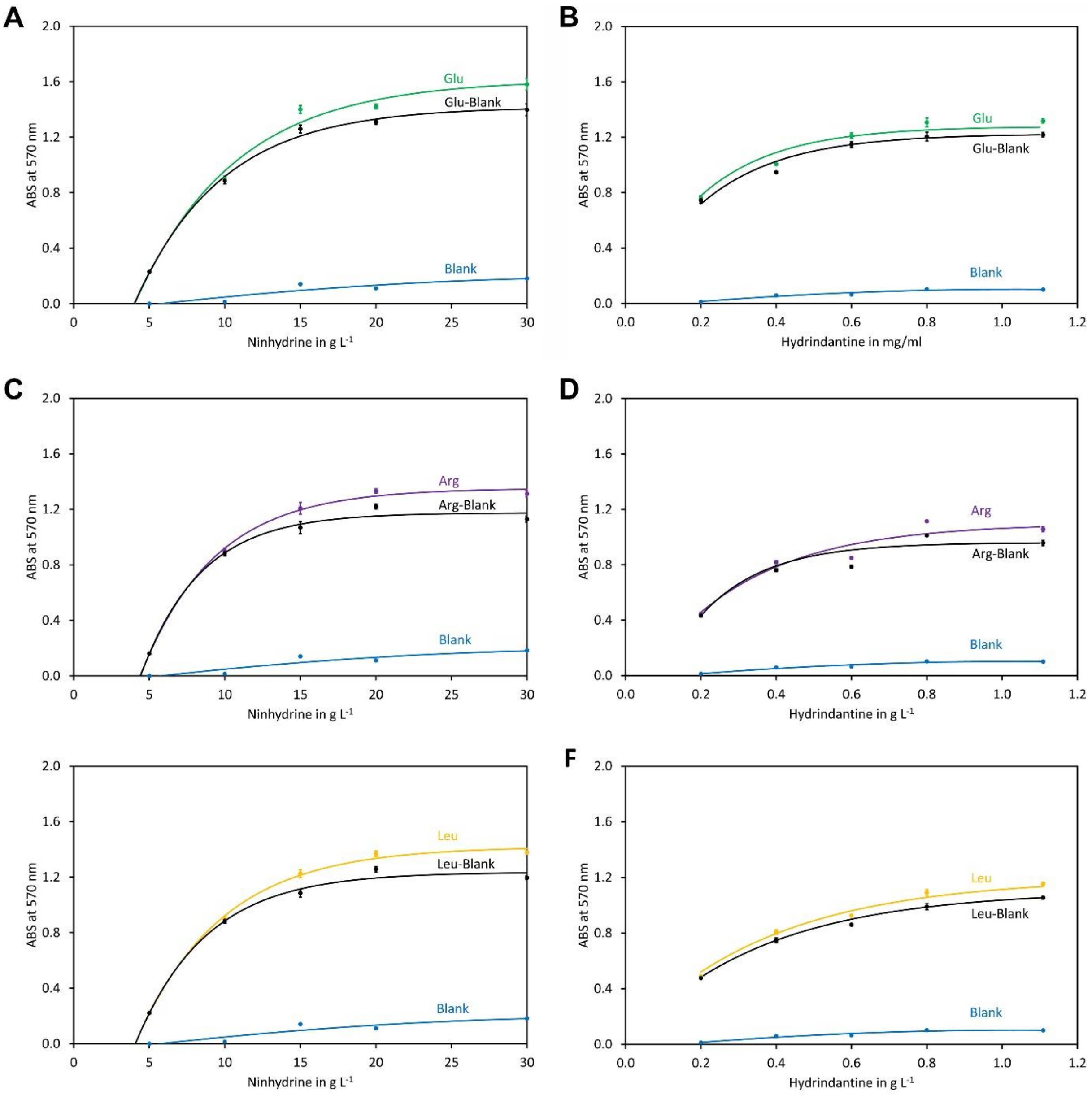

Supplementary Figure S3: Impact of the ninhydrin (A, C, E) and hydrindantin concentrations (B, D, F) on the reaction with glutamic acid (A, B), arginine (C, D) and leucine (E, F). For (A, C, E) the indicated concentration of ninhydrin was used, and hydrindantin was added at a ratio of hydrindantin/ninhydrin of 0.01/1. For (B, D, F) ninhydrin was used at a fixed concentration of 20 g L^{-1} and hydrindantin was added at the indicated concentration. Acetic acid was used at a final concentration of 1.6 mol L^{-1} and potassium acetate at 0.8 mol L^{-1}. The mixture contained DMSO and aqueous acetate buffer at a ratio of 40/60 (v/v).
