# Supplementary material for: The Ninhydrin Reaction Revisited: Optimisation and Application for Quantification of Free Amino Acids
Source: Molecules. 2024 Jul 10;29(14):3262. doi: 10.3390/molecules29143262 (PMC11278723; doi:10.3390/molecules29143262)
Supplement: Supplementary file 1 [file molecules-29-03262-s001.zip › Supplementary Figure S4.pdf]

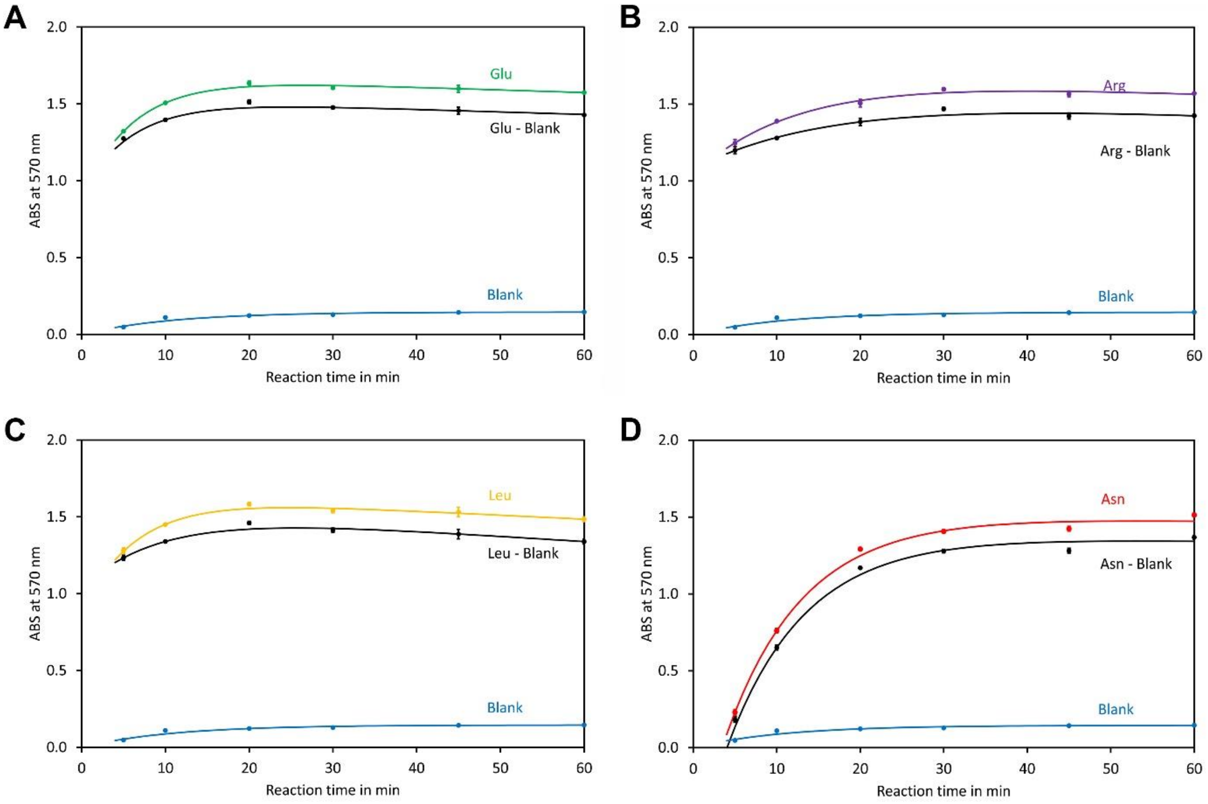

Supplementary Figure S4: Reaction kinetics for glutamic acid, arginine, leucine, and asparagine. (**A**) Using the optimised reagent, for each time point 4 independent reactions were heated to 90°C. Subsequently, the tubes were immediately transferred to cold water to cool them to room temperature to stop the reaction. The final concentration of glutamic acid was 0.5 mmol L^{-1}. Similar experiments were performed for (**B**) arginine and (**C**) leucine. Data obtained with asparagine (**D**) are shown for comparison. The data points and error bars represent the averages and standard deviations of four independent reactions, respectively.
