# Supplementary material for: The Ninhydrin Reaction Revisited: Optimisation and Application for Quantification of Free Amino Acids
Source: Molecules. 2024 Jul 10;29(14):3262. doi: 10.3390/molecules29143262 (PMC11278723; doi:10.3390/molecules29143262)
Supplement: Supplementary file 1 [file molecules-29-03262-s001.zip › Supplementary Figure S5.pdf]

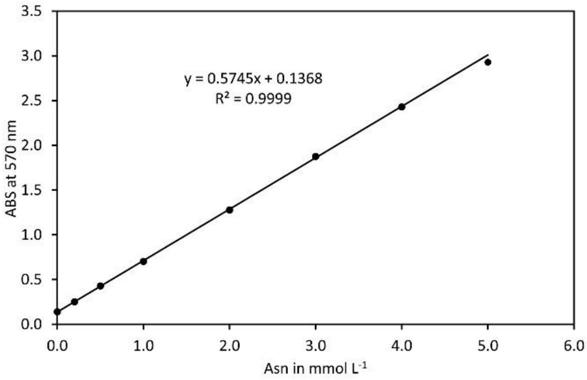

Supplementary Figure S5: Calibration curve obtained with the optimised reagent and reaction conditions. Each standard (200 µL) was mixed with 800 µL of the optimised reagent and heated to 90°C for 45 min. Then, 500 µL of each reaction mixture was diluted with 2500 µL 2-propanol/water = 1/1 (v/v). The asparagine concentration refers to that of the standard. The standard with a concentration of 5 mmol L^{-1} was out of the linear rage and thus omitted from the calibration curve.
