# Supplementary material for: The Ninhydrin Reaction Revisited: Optimisation and Application for Quantification of Free Amino Acids
Source: Molecules. 2024 Jul 10;29(14):3262. doi: 10.3390/molecules29143262 (PMC11278723; doi:10.3390/molecules29143262)
Supplement: Supplementary file 1 [file molecules-29-03262-s001.zip › Supplementary Methods S1.pdf]

Supplementary Methods S1: Step-by-step protocol for quantification of free amino acids.

Chemicals

Ninhydrin (purity >99%) CAS number: 485-47-2
Hydrindantin (purity >97%) CAS number: 5103-42-4
Acetic acid (purity >99.5%) CAS number: 64-19-7
Potassium acetate (purity >99%) CAS number: 127-08-2
Dimethyl sulfoxide, DMSO (purity >99.8%) CAS number: 67-68-5
2-Propanol (purity >99.8%) CAS number: 67-63-0
Asparagine monohydrate (purity >99%) CAS number: 5794-13-8

Reagents

Acetic acid/potassium acetate buffer (dissolve 98.1 g potassium acetate in approximately 250 mL water prior to addition of 111 mL acetic acid. Add water to 500 ml. The buffer is stable at room temperature for at least one year)

Ninhydrin reagent (weigh 550±10 mg ninhydrin and 22±2 mg hydrindantin into a beaker and add 11 mL DMSO. Mix gently until the solid has dissolved completely. Subsequently, add 11 mL acetic acid/potassium acetate buffer and mix well. The reagent should be used within 12 h)

2-Propanol/water = 1/1 (v/v) (mix 500 mL 2-propanol with 500 mL water)

Asparagine 2.5 mmol L^{-1} (transfer 375.4±0.5 mg asparagine monohydrate into a 1 L volumetric flask, dissolve in water and add water to the mark. The solution can be kept at -20°C for at least one year)

Method

1) Prepare standards by mixing the following solutions in 1.5 mL reaction tubes. The obtained asparagine concentrations are given in the second column.

Standard no.	Asparagine concentration mmol L^{-1}	Volume of distilled water µL	Volume of asparagine 2.5 mmol L^{-1} µL
1	0	200	0
2	0.1	192	8
3	0.2	184	16
4	0.5	160	40
5	1	120	80
6	2.5	0	200

2) Transfer 200 µl of each sample into 1.5 mL reaction tubes.
3) Add to each tube (standards and samples) 800 µL ninhydrin reagent and mix very well.
4) Incubate the samples in a block thermostat set to 90°C for 45 min.
5) Take the tubes out and let them cool to room temperature.
6) Transfer 500 µL of each reaction into 3 ml macro cuvettes (1 cm optical path).
7) Add 2500 µL 2-propanol/water = 1/1 (v/v) and mix well.
8) Measure the absorbance at 570 nm.
