# Supplementary material for: The Ninhydrin Reaction Revisited: Optimisation and Application for Quantification of Free Amino Acids
Source: Molecules. 2024 Jul 10;29(14):3262. doi: 10.3390/molecules29143262 (PMC11278723; doi:10.3390/molecules29143262)
Supplement: Supplementary file 1 [file molecules-29-03262-s001.zip › Supplementary Table S1.pdf]

Supplementary Table S1. pH of aqueous acetic acid/potassium acetate buffers.

Acetic acid mol L^{-1}	Potassium acetate mol L^{-1}	pH undiluted buffer	pH buffer/water = 40/60 (v/v)	pH buffer/water/DMSO = 40/20/40 (v/v/v)	pH buffer/water = 1/24 (v/v)	pH theoretical[c]
0	2	8.69	8.29	9.59	7.68	-
1	2	5.50	5.19	6.47	4.97	5.05
2	2	5.24	4.93	6.18	4.69	4.75
4[a]	2[a]	4.93	4.58	5.88[b]	4.33	4.45
6	2	4.76	4.42	5.71	4.17	4.27

[a] Composition of the optimised undiluted acetic acid/potassium acetate buffer.

[b] This composition corresponds to the optimised reaction conditions.

[c] pH calculated according to the Henderson-Hasselbalch equation.
